# Impact of the Changes in the Frequency of Social Participation on All-Cause Mortality in Japanese Older Adults: A Nationwide Longitudinal Study

**DOI:** 10.3390/ijerph19010270

**Published:** 2021-12-27

**Authors:** Keiichi Shimatani, Mayuko T. Komada, Jun Sato

**Affiliations:** Division of Nursing, Higashigaoka Faculty of Nursing, Tokyo Healthcare University, Tokyo 152-8558, Japan; m-komada@thcu.ac.jp (M.T.K.); j-satoh@thcu.ac.jp (J.S.)

**Keywords:** social participation, social activities, social capital, mortality, well-being, older adults

## Abstract

Previous studies have shown that more frequent social participation was associated with a reduced risk of mortality. However, limited studies have explored the changes in the frequency of social participation in older adults. We investigated the impact of the changes in the frequency of social participation on all-cause mortality in Japanese older adults aged 60 years and older. The current study, conducted as a secondary analysis, was a retrospective cohort study using open available data. The participants were 2240 older adults (45.4% male and 54.6% female) sampled nationwide from Japan who responded to the interview survey. Changes in the frequency of social participation were categorized into four groups (none, initiated, decreased, and continued pattern) based on the responses in the baseline and last surveys. The Cox proportional-hazards model showed a decreased risk of all-cause mortality in decreased and continued patterns of social participation. Stratified analysis by sex showed a decreased risk of mortality in the continued pattern only among males. The results of the current study suggest that the initiation of social participation at an earlier phase of life transition, such as retirement, may be beneficial for individuals.

## 1. Introduction

Social capital is an important public health approach for the maintenance and health promotion of older adults considering the aging society worldwide. Previous studies have reported that social participation in older adults, at the individual level in community organizations, was a social capital that led to a reduction in mortality [1,2,3]. Furthermore, recent findings have suggested that it reduced the risk of functional [4,5] and cognitive impairment [6,7,8,9] and psychological distress [10,11], in addition to reduced mortality risk.

Several reports have investigated the frequency of social participation in health maintenance, such as for reducing mortality risk. A population-based study in Finland, on Finnish men and women aged 30–59 years, observed that more frequent participation in leisure activities compared to less frequent participation contributed to reduced all-cause mortality risk [12]. Nieminen et al. explored the association between the three aspects of social capital (social support, social participation, and trust) and mortality risk. After they adjusted for several sociodemographic factors, health behaviors, and health and biological risk factors, they found that more non-active social participation was associated with an increased risk of mortality [13]. The Nord-Trøndelag Health Study (HUNT), a longitudinal study in Norway, reported that the frequency of social participation of less than 1, 1 to less than 2, and 2 or more times per week reduced mortality risk by 18%, 31%, and 39%, respectively [14]. Participation in both receptive and creative activities at least twice a week was associated with a 29% reduction in cancer-related mortality [15]. Fancourt et al. studied the association of all-cause mortality among adults aged 50 years and older and reported that those who participated in receptive arts activities at least once every few months or more had a 31% lower mortality rate compared to those who never participated [16]. These previous findings suggested that more frequent participation was associated with a reduced mortality risk and that a system should be designed to maintain participation once it has been initiated [17].

However, findings on the level of social participation remain unclear. Since the previous studies used only information from the baseline survey, they assumed that the current status of social participation from the time of the baseline survey continued. Thus, behavioral changes over time have not been fully assessed and examined. Exploring the association between changes in the frequency of social participation and mortality risk can provide and extend knowledge of when it is most appropriate to approach older adults. Specifically, to reveal whether maintaining the frequency of social participation is still recommended, as in previous studies, or whether reducing the frequency is also acceptable, or whether even initiating social participation by changing those habits in the aging generation is effective in reducing mortality risk. These findings will be valuable in promoting health policies for older adults.

In the current study, we conducted a longitudinal secondary analysis, based on the data obtained from a national survey of Japanese people aged 60 years and older, to investigate the impact of the changes in the frequency of social participation on all-cause mortality in older adults.

## 2. Materials and Methods

### 2.1. Study Design and Population

This study was conducted as a retrospective cohort study using available open data. The data for secondary analysis were obtained from a national survey of Japanese older adults and were provided by the Social Science Japan Data Archive, Center for Social Research and Data Archives, Institute of Social Science, the University of Tokyo [18].

The baseline survey (Wave 1) was conducted in 1987 among a stratified random sample of men and women aged 60 years and older from across Japan, with follow-up surveys conducted every three years in 1993 (Wave 3), 1999 (Wave 5), 2002 (Wave 6), and finally in 2006 (Wave 7), with added samples aged 60–62 in 1990 (Wave 2) and 60–65 in 1996 (Wave 4). The survey area was divided into 11 regional blocks (Hokkaido, Tohoku, Kanto, Hokuriku, Higashiyama, Tokai, Kinki, Chugoku, Shikoku, Kitakyushu, and Minami-Kyushu), located nationwide, and the survey points were selected using the standard survey areas of the National Census as the primary extraction unit. The secondary extraction units were individuals selected by an equally spaced sampling method based on the Basic Resident Ledger (or the Electoral Roll if the Basic Resident Ledger was not available). Participants were interviewed by a researcher at their homes. For the second and subsequent surveys, when participants were unavailable to respond either because of illness or other reasons, the questionnaires were completed by another family member who provided information regarding family arrangements and health conditions.

We defined the observed period of follow-up as 11 years and included a total of 3990 respondents from each baseline survey in 1986, 1990, and 1996. To observe changes in the frequency of social participation, respondents who did not participate in the second and subsequent surveys (N = 614) and those who lacked information on the frequency of social participation throughout the surveys (N = 403), were excluded. Furthermore, since mortality as an outcome was strongly associated with age, we excluded participants aged 70 years or older (N = 733), after considering the observation period of this study and as the average life expectancy of Japanese people in 1990 was 78.91 years [19]. Thus, the final analysis included 2240 older adults (male = 1018, female = 1222) aged 60–69 years.

### 2.2. Measurement

#### 2.2.1. All-Cause Mortality

All-cause mortality data were based on information obtained by confirming the resident card. In cases where the resident card could not be confirmed, due to the absence of the corresponding person at the listed address or lack of cooperation from the local government, information was obtained from the family members. If no information on the date of death was obtained from family members, etc., the time until survival was estimated as “unknown” and included as a censored case.

#### 2.2.2. Frequency of Social Participation

The frequency of social participation was assessed using the questionnaire item “how many times do you go out to meetings, clubs, or groups, such as neighborhood associations, community associations, senior citizen clubs, business associations, or religious groups?” The responses included “twice a week”, “once a week”, “2 or 3 times a month”, “about once a month”, “less than once a month” and “never”. We further grouped them into four categories: 0, less than 1, 1–3, 4 or more times per month.

#### 2.2.3. Covariates

We included other covariates such as age, sex, body mass index (BMI) (<18.5, 18.5–25.0, ≥25.0), education (≤6, 7–12, ≥13 years), marital status (married, single/divorced/separated/died), working status (none, working, housework), living arrangements (living alone, with another, or with more than two people), and annual total household income (≤3,000,000, 3,000,000–5,000,000, 5,000,000–10,000,000, ≥10,000,000 JPY) as social economics status.

To consider the health status of the participants, smoking status (never or current/former), alcohol consumption (non-drinker or drinker), self-reported health (good, normal, poor), history of hospitalization within six months, and number of comorbidities were also addressed as covariates. Comorbidities were defined as a history of hypertension, stroke, diabetes, respiratory disease, Parkinson’s disease, bone fracture, joint disease, cardiovascular disease, digestive disease, renal disease, anemia, ophthalmologic disease, skin disease, back pain, quadriplegia, circulatory disturbance of the extremities, and psychiatric disease. The number of comorbidities was treated as a continuous variable.

### 2.3. Statistical Analysis

**Figure 1:**
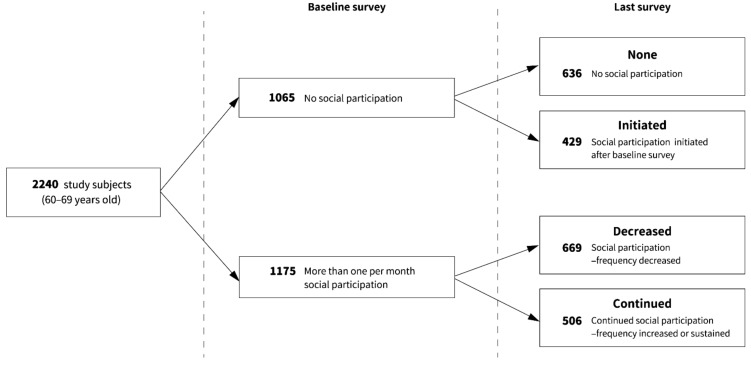
Flow diagram of the frequency pattern of social participation in the study participants.

Participants who had no social participation in the baseline survey and did not participate in the last survey were defined as the none pattern, whereas those whose frequency of social participation increased from 0 were classified as the initiated pattern. Furthermore, we divided participants who had social participation at least once a month in the baseline survey into two categories: the decreased pattern—those who had decreased in frequency in the last survey, and the continued pattern—those who continued or increased their participation in the last survey (Figure 1).

The characteristics of the study participants were compared using a chi-square test for four groups of social participation: none, initiated, decreased, and continued patterns. After the mortality rate was described, considering the background characteristics of the study participants, we used a multivariate Cox proportional-hazards model to estimate the hazard ratios (HRs) and 95% confidence intervals (CIs) of all-cause mortality according to changes in the frequency of social participation. Furthermore, we used Aalen’s linear hazards model to estimate how the effect of changes in the frequency of social participation differs over time [20]. In the multivariate-adjusted model, we adjusted for all the covariates to control for confounding factors such as age, sex, BMI, duration of education, marital status, working status, living arrangement, annual total household income, smoking status, alcohol consumption, self-reported health, hospitalization within six months, and the number of comorbidities.

The percentage of missing values across the six variables varied between 0.1 and 13.6%. In total, 391 of the 2240 participants (17.5%) had incomplete responses. Epidemiological studies suggest the use of multiple imputations (MI), a principal method in epidemiological studies, to obtain estimates closer to the “true” full data effect [21,22]. To impute the missing data, we carried out a multivariate imputation using chained equations (MICE) to handle missing data and create and analyze 20 multiply imputed datasets. Incomplete variables were imputed using all the covariates: age, sex, marital status, living arrangements, smoking status, alcohol consumption, history of hospitalization within six months, and the frequency of social participation at the baseline and last survey after the Nelson Aalen estimator of cumulative hazard was calculated and included. The calculation was estimated with multiple regression applied to each imputed dataset separately using Rubin’s rules.

Statistical significance was set at *p* < 0.05. All calculations and statistical analyses were performed using Stata software (version 17.1 SE; Stata Corp., College Station, TX, USA).

## 3. Results

Table 1 shows the all-cause mortality rates considering the background characteristics of the respondents over the 11-year observation period. During the follow-up period for 10,561.6 person-years for men and 12,602.1 person-years for women, 127 and 76 deaths were observed, respectively. Regarding the frequency of social participation in the initial survey, the mortality rates were 10.3 and 6.6 per 1000 person-years for 0 times per month and ≥4 times per month, respectively. Regarding the frequency of social participation in the last survey, the mortality rates were 10.1 and 4.0 per 1000 person-years for 0 times per month ≥4 times per month, respectively. The mortality rates were lower for respondents who participated more frequently in social activities in both surveys.

The number of respondents in each change pattern of the frequency of social participation (none, initiated, decreased, and continued pattern) were 636, 429, 669, and 506, respectively (Table 2). The distribution of covariates was compared between these groups using Chi-squared tests. Results revealed statistically significant differences in the duration of education (*p* = 0.021, Cramér’s V = 0.054), smoking status (*p* < 0.001, Cramér’s V = 0.090), alcohol consumption (*p* < 0.001, Cramér’s V = 0.098), and self-reported health (*p* < 0.001, Cramér’s V = 0.085).

The HRs for all-cause mortality for those with each change pattern of the frequency of social participation were estimated after imputing the MICE (Table 3). In the multivariate-adjusted model, the HR of the initiated pattern was 0.84 (95% CI 0.56–1.24) compared to those with none pattern. In the initiated pattern, the HR was lower than 1.00, however, the difference was not statistically significant. The HRs of the decreased and continued patterns were significantly lower at 0.70 (95% CI 0.48–0.99) and 0.63 (95% CI 0.41–0.94), respectively. Furthermore, the HRs for all-cause mortality with each change pattern of the frequency of social participation, male and female, were separately estimated. The HRs for males showed a statistically significant reduction in risk at 0.50 (95% CI 0.28–0.85) only for the continued pattern. The HRs for females were not significantly different from none pattern, regardless of the change pattern of frequency of social participation, even in the multivariate models.

**Figure 2:**
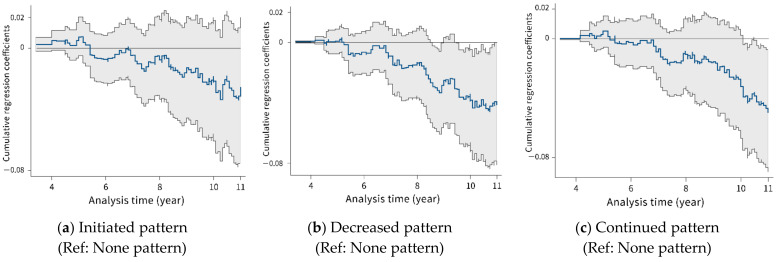
Plot of the estimated cumulative regression coefficient for the changes in the frequency of the pattern of social participation with a 95% confidence interval based on Aalen’s linear hazards model *: (**a**) Initiated pattern; (**b**) Decreased pattern; (**c**) Continued pattern. * Performed a multivariate model before imputing the MICE.

The Aalen’s cumulative regression coefficients for changes in the pattern of frequency of social participation are plotted in Figure 2. The coefficients were estimated with reference to none pattern. Participants included in the analysis were those who participated in the second and subsequent surveys conducted every three years following the baseline survey. Therefore, cases of mortality within three years were excluded, which resulted in the cumulative regression coefficients, calculated after three years of follow-up. Based on Figure 2a, the trend of cumulative coefficients for the initiated pattern was nearly linear with a slight negative slope; over the study period of approximately nine years, the curve got lower than the zero line. The plots indicating the trend for decreased and continued patterns were similar to those for the initiated pattern; however, time taken for the slope to get steeper was approximately seven years (Figure 2b,c). The initiated pattern may have a late effect compared to decreased and continued patterns. The upper 95% confidence band in decreased and continued patterns lies under the zero line after 11 years, suggesting that there were significant differences compared to none pattern. In other words, since the zero line was within 95% CI, the initiated pattern was not significant.

## 4. Discussion

The current study investigated the impact of changes in the frequency of social participation on the risk of all-cause mortality in a retrospective cohort of a nationwide survey among Japanese older adults. Few studies have been reported on this issue. Our main finding was that continued or decreased frequency of social participation was associated with a decreased risk of all-cause mortality. However, regarding social participation initiated after the age of 60 years, there was no such association. Furthermore, a decreased risk of all-cause mortality with a pattern of change in the frequency of social participation was observed only in male participants.

A 10-year longitudinal study showed that the odds ratio of poor self-reported health increased significantly after 10 years of no/low social participation. Additionally, the negative effects of a loss of social participation were observed in both sexes [23]. A report which examined the relationship between the changes in social capital and depression observed that changes in social capital were important in the prevention of depression [24]. Based on these reports and findings that self-related health and depressive symptoms increased the risk of mortality [25,26,27], continued social participation may prevent depressive symptoms and other health problems, maintain health, and provide a protective effect against mortality.

The effects of social participation on health are mediated by social support and a sense of social cohesion [28]. Increased social support provides individuals with access to various types of support and assistance when needed. Social cohesion refers to the sense of trust and reciprocity that individuals have in their communities. The more social cohesion, the more likely individuals are to trust people in their community and to recognize that they will help them when in need of assistance [28]. These influences may promote the diffusion of health information or establishment of healthy behavioral norms, which in turn could lead to health behaviors [29,30]. Several recent reports have documented an association between social participation and health behaviors. Social participation has been positively associated with health behaviors, such as smoking cessation (low smoking rate) [31], excessive alcohol consumption, leisure-time physical activity, and adequate sleep duration [32]. It may also promote healthier dietary behaviors, such as increased fruit and vegetable intake, regardless of the sociodemographic status [33].

Furthermore, according to a recent report, social participation was identified as moderately protective for the onset of specific non-communicable diseases (NCDs), hypertension [34,35], diabetes, and stroke, in middle-aged and older adults [36]. The risk factors for NCDs, such as systolic and diastolic blood pressure (BP), BMI, total cholesterol (TC), fasting blood glucose (FBS) levels, and blood plasma levels of glycosylated hemoglobin (HbA1c), were considered as indicators of the cumulative biological risk (CBR) of allostatic load [37,38,39]. Health behaviors and lifestyles that avoid increasing the CBR are essential to prevent NCDs and require long-term maintenance rather than temporary interventions. Although the mechanisms between social participation and health behaviors are unclear, the preventive effect of social participation may require cumulative, small changes in individual behavior, that is, changes in behavior for a specific length of time, to allow modification of health behaviors.

In the present study, no decreased risk of mortality was observed among older adults who initiated social participation after the age of 60 years. Furthermore, the effects of the initiated pattern may be delayed compared to the decreased or continued pattern. This finding indicates that the potential positive impact on mortality risk reduction from exposure to social participation at the individual level is not only dose-dependent with frequency but may also take a certain length of time. This suggests that a time-dependent aspect of continued exposure to social participation may contribute to this effect.

Regarding the target age for a greater preventive effect of social participation, it has been reported that the effect on health outcomes was greater for those aged 60–69 years compared to those from younger or older age groups [40]. Furthermore, older adults in their early 60s may see a beneficial effect from social participation on the onset of dementia [41]. A factor that increases depressive symptoms and the magnitude of psychological stress is retirement, which tends to occur in their early 60s, suggesting that retirement age may increase depressive symptoms among older adults in Japan, especially among men in lower occupational classes [42,43]. It was also reported that the transition to retirement for many Japanese workers involved a disconnection from work colleagues and a loss of meaning in their lives. Therefore, social participation could be a prescription that could mitigate the potential negative impacts of retirement by covering for such losses [43]. The participants of this study were in their 60s around 1990; moreover, this generation of Japanese workers was a cohort with guaranteed lifetime employment and a strong commitment to their companies, which is consistent with the background of participants from previous studies. In our results, social participation was also associated with a decreased risk of mortality in men. Our results and previous findings on the association between depression and increased risk of mortality [26,27] have suggested that social participation contributed to a decreased risk of mortality in Japanese men who were more likely to experience a retirement event in their 60s. For women, there was no association between changes in frequency of social participation and a decreased risk of mortality. The average life expectancy of Japanese women around 1990 was 81.9 years [19]. We should also consider the fact that even though the participants in their 60s were followed for 11 years, they were observed only up to 80 years of age, which may have influenced the results.

To the best of our knowledge, this is the first study to investigate how changes in the frequency of social participation in older adults contributed to mortality risk using population data representative of the Japanese population. However, this study has several limitations. First, the frequency of social participation before the baseline survey was unknown, which may have caused misclassification when stratifying changes in the frequency of social participation. Since we observed the changes from the baseline, if the participants answered that they were not participating in social activities at the time of the baseline survey, they were classified into the group that was “none pattern” or “initiated pattern” in social participation. Even if they participated in social activities a while before the time of the survey, some participants may have responded with their habits at the time of the survey. According to the distribution of the frequency of social participation among patients who died within three years, 65% who died did not participate in social activities (data not shown). Therefore, the HRs estimated in this study may have been underestimated. Second, the types of groups in which the respondents participated in social activities were not available. Thus, we could not consider them in detail. Several studies have reported that patterns of social participation prevent the development of functional disability and instrumental activities of daily living (IADL) decline in sports clubs and cultural/hobby clubs [5,44,45]. Participation in hobby/sports groups reduced the risk of mortality, while participation in religious groups increased the risk of mortality and showed differences in mortality and other prognostic factors [46]. Third, for the statistical analysis, some potential confounding factors were not adjusted for. For example, we could not address activities of daily living (ADL), known to contribute to mortality risk, as a confounding factor due to the different number of research items in each wave of the survey. However, we adjusted for factors such as the number of comorbidities, history of hospitalization within six months, and personal health perspective to remove confounding factors whenever possible. Future research requires longitudinal studies that integrate detailed information on the types of social participation groups and the effects of age.

## 5. Conclusions

The results of the current study suggested that initiated social participation after the age of 60 years had a limited preventive effect on all-cause mortality risk, especially among men. These findings suggest that public health policies that contribute to the health of older adults should be designed to maintain social participation once it has been initiated. Furthermore, the initiation of social participation at an earlier phase of life transition, such as retirement, may be beneficial for individuals.

## Figures and Tables

**Table 1:** Mortality rates by background characteristics of the study participants (N = 2240).

Variables	Number (%)	Incidence/Person Year	Incidence Rate/1000 Person-Years
Sex Male	1018 (45.4)	127/10,561.6	12.0
Female	1222 (54.6)	76/12,602.1	6.0
Age (years) 60–64	1621 (72.4)	134/16731.9	8.0
65–69	619 (27.6)	69/6431.8	10.7
BMI ^1^ <18.5	162 (7.2)	21/1657.3	12.7
18.5–25.0	1629 (72.7)	149/16,862.5	8.8
≥25.0	447 (20.0)	32/4626.0	6.9
Missing	2 (0.1)	1/17.8	56.1
Duration of education (years)			
≤6	257 (11.5)	28/2696.6	10.4
7–12	1727 (77.1)	156/17,793.3	8.8
≥13	239 (10.7)	18/2499.4	7.2
Missing	17 (0.8)	1/174.4	5.7
Marital status			
Married	1769 (79.0)	166/18,258.3	9.1
Single, divorced, separated, died	471 (21.0)	37/4905.4	7.5
Working status			
None	585 (26.1)	81/6042.6	13.4
Working	1023 (45.7)	84/10,570.9	7.9
Housework	630 (28.1)	38/6528.2	5.8
Missing	2 (0.1)	0/43.0	0.0
Living arrangement			
Living alone	153 (6.8)	11/1604.1	6.9
With another	841 (37.5)	78/8642.4	9.0
Or with more than two people	1246 (55.6)	114/12,917.2	8.8
Total household income (JPY)			
≤3,000,000	798 (35.6)	88/8349.8	10.5
3,000,000–5,000,000	489 (21.8)	42/5125.8	8.2
5,000,000–10,000,000	437 (19.5)	34/4474.1	7.6
≥10,000,000	212 (9.5)	11/2227.3	4.9
Missing	304 (13.6)	28/2986.8	9.4
Smoking status			
Current/Former-smoking	827 (36.9)	108/8652.6	12.5
Never	1413 (63.1)	95/14,511.1	6.5
Alcohol consumption			
Drinker	1032 (46.1)	108/10,674.0	10.1
Non-drinker	1208 (53.9)	95/12,489.7	7.6
Self-reported health			
Good	1173 (52.4)	103/12,199.5	8.4
Normal	820 (36.6)	70/8415.6	8.3
Poor	234 (10.5)	27/2411.2	11.2
Missing	13 (0.6)	3/137.4	21.8
Hospitalization within six months			
No	2136 (95.4)	194/22,060.8	8.8
Yes	104 (4.6)	9/1102.8	8.2
No of comorbidities			
0	940 (42.0)	73/9782.8	7.5
1	754 (33.7)	78/7733.8	10.1
≥2	476 (21.3)	46/4933.2	9.3
Missing	70 (3.1)	6/714.0	8.4
Frequency of social participation at baseline survey (times/month)			
0	1065 (47.5)	112/10,896.1	10.3
<1	383 (17.1)	29/4038.0	7.2
1–3	455 (20.3)	39/4768.4	8.2
≥4	337 (15.0)	23/3461.2	6.6
Frequency of social participation at last survey (times/month)			
0	989 (44.2)	102/10,086.0	10.1
<1	414 (18.5)	35/4314.8	8.1
1–3	506 (22.6)	52/5235.3	9.9
≥4	331 (14.8)	14/3527.6	4.0

^1^ BMI, body mass index.

**Table 2:** Background characteristics of the study participants by changes in the frequency of the pattern of social participation.

	Changes in the Frequency of the Pattern of Social Participation					
	None Pattern (N = 636)	Initiated Pattern (N = 429)	Decreased Pattern (N = 669)	Continued Pattern (N = 506)	*p* Value	Effect Size
Sex					0.704	0.025
Male	280 (44.0)	197 (45.9)	315 (47.1)	226 (44.7)		
Female	356 (56.0)	232 (54.1)	354 (52.9)	280 (55.3)		
Age (years)					0.211	0.045
60–64	476 (74.7)	316 (73.7)	468 (70.0))	361 (71.3)		
65–69	160 (25.2)	113 (26.3)	201 (30.0)	145 (28.7)		
BMI ^1^					0.227	0.042
<18.5	55 (8.7)	28 (6.5)	54 (8.1)	25 (4.9)		
18.5–25.0	461 (72.5)	322 (75.1)	470 (70.3)	376 (74.3)		
≥25.0	120 (18.9)	78 (18.2)	144 (21.5)	105 (20.8)		
Missing	0 (0.0)	1 (0.2)	1 (0.2)	0 (0.0)		
Duration of education (years)					0.021	0.054
≤6	100 (15.7)	48 (11.2)	66 (9.9)	43 (8.5)		
7–12	474 (74.5)	327 (76.2)	526 (78.6)	400 (79.1)		
≥13	57 (9.0)	50 (11.7)	73 (10.9)	59 (11.7)		
Missing	5 (0.8)	4 (0.9)	4 (0.6)	4 (0.8)		
Marital status					0.123	0.050
Married	486 (76.4)	334 (77.9)	546 (81.6)	403 (79.6)		
Single, divorced, separated, died	150 (23.6)	95 (22.1)	123 (18.1)	103 (20.4)		
Working status					0.506	0.035
None	180 (28.3)	115 (26.8)	177 (26.5)	113 (22.3)		
Working	275 (43.2)	190 (44.3)	312 (46.6)	246 (48.6)		
Housework	180 (28.3)	124 (28.9)	179 (266.8)	147 (29.1)		
Missing	1 (02)	0 (0.0)	1 (0.2)	0 (0.0)		
Living arrangement					0.274	0.041
Living alone	54 (8.5)	26 (6.1)	42 (6.3)	31 (6.1)		
With another	224 (35.2)	152 (35.4)	262 (39.2)	203 (40.1)		
Or with more than two people	358 (56.3)	251 (58.5)	365 (54.6)	272 (53.8)		
Total household income (JPY)					0.053	0.056
≤3,000,000	247 (38.8)	147 (34.3)	233 (34.8)	171 (33.8)		
3,000,000–5,000,000	130 (20.4)	100 (23.3)	153 (22.9)	106 (21.0)		
5,000,000–10,000,000	102 (16.0)	77 (18.0)	146 (21.8)	112 (22.1)		
≥10,000,000	54 (8.5)	42 (9.8)	65 (9.7)	51 (10.1)		
Missing	103 (16.2)	63 (14.7)	72 (10.8)	66 (13.0)		
Smoking status					<0.001	0.090
Current/Former-smoking	362 (56.9)	289 (67.4)	420 (62.8)	342 (67.6)		
Never	274 (43.1)	140 (32.6)	249 (37.2)	164 (62.4)		
Alcohol consumption					<0.001	0.098
Drinker	246 (38.7)	203 (47.3)	341 (51.0)	242 (47.8)		
Non-drinker	390 (61.3)	226 (52.7)	328 (49.0)	264 (52.2)		
Self-reported health					<0.001	0.085
Good	298 (46.9)	229 (53.4)	366 (54.7)	280 (55.3)		
Normal	227 (35.7)	159 (37.1)	240 (35.9)	194 (38.3)		
Poor	109 (17.1)	37 (8.6)	58 (8.7)	30 (5.9)		
Missing	2 (0.3)	4 (0.9)	5 (0.8)	2 (0.4)		
Hospitalization within six months					0.734	0.024
No	603 (94.8)	412 (96.0)	636 (95.1)	485 (95.9)		
Yes	33 (5.2)	17 (4.0)	33 (4.9)	21 (4.2)		
No of comorbidities					0.286	0.040
0	247 (38.8)	204 (47.6)	272 (40.7)	217 (42.9)		
1	215 (33.8)	133 (31.0)	234 (35.0)	172 (34.0)		
≥2	152 (23.9)	81 (18.9)	140 (20.6)	103 (20.4)		
Missing	22 (3.5)	11 (2.6)	23 (3.4)	14 (2.8)		

^1^ BMI, body mass index.

**Table 3:** Hazard ratios and 95% confidence intervals of all-cause mortality according to changes in the frequency of the pattern of social participation after imputing the MICE ^1^.

	Person-Years	HR ^2^1 ^a^	(95% CI ^3^)	HR ^2^2 ^b^	(95% CI ^3^)
Over all (N = 2240)					
The pattern of social participation					
None pattern	602.9/6429.9	1.00	-	1.00	-
Initiated pattern	332.2/4466.2	0.77 *	(0.53–1.14)	0.84 *	(0.56–1.24)
Decreased pattern	461.6/6956.6	0.66 *	(0.46–0.93)	0.70 *	(0.48–0.99)
Continued pattern	281.0/5311.0	0.57 *	(0.38–0.85)	0.63 *	(0.41–0.94)
Male (N = 1018)					
The pattern of social participation					
None pattern	404.4/2813.4	1.00	-	1.00	-
Initiated pattern	189.4/2073.4	0.65	(0.39–1.07)	0.77	(0.46–1.28)
Decreased pattern	298.3/3283.3	**0.59**	(0.38–0.90)	0.66	(0.42–1.01)
Continued pattern	149.5/2391.5	**0.43**	(0.25–0.72)	**0.50**	(0.28–0.85)
Female (N = 1222)					
The pattern of social participation					
None pattern	198.5/3616.5	1.00	-	1.00	-
Initiated pattern	142.8/2392.8	1.10	(0.59–2.06)	1.20	(0.62–2.27)
Decreased pattern	163.3/3673.3	0.80	(0.43–1.47)	0.82	(0.43–1.52)
Continued pattern	131.5/2919.5	0.89	(0.47–1.66)	0.94	(0.49–1.78)

^1^ MICE, Multiple imputation by chained equation; ^2^ HR, Hazard ratio; ^3^ CI, confidential interval; a: Adjusted for age. b: Adjusted for age, BMI (<18.5, 18.5–24.9, ≥25.0), duration of education (≤6, 7–12, ≥13 years), marital status (married, single/divorced/separated/died), working status (none, working, housework), living arrangement (living alone, with another, or with more than two people), total household income (≤3,000,000, 3,000,000–5,000,000, 5,000,000–10,000,000, ≥10,000,000), smoking (current/former, never), alcohol consumption (drinker, never), self-reported health (good, normal, poor), hospitalization within six months (no, yes), no of comorbidities (continuous). *Added sex for adjustment. Bold type is statistically significant.

## Data Availability

The data for this secondary analysis, name of the survey and the name of depositor, was provided by the Social Science Japan Data Archive, Center for Social Research and Data Archives, Institute of Social Science, The University of Tokyo. The following are available online at https://csrda.iss.u-tokyo.ac.jp/english/ (accessed on 18 October 2021).
